# Gut microbiota in patients with kidney stones: a systematic review and meta-analysis

**DOI:** 10.1186/s12866-023-02891-0

**Published:** 2023-05-19

**Authors:** Tianhui Yuan, Yuqi Xia, Bojun Li, Weimin Yu, Ting Rao, Zehua Ye, Xinzhou Yan, Baofeng Song, Lei Li, Fangyou Lin, Fan Cheng

**Affiliations:** Department of Urology, Renmin Hospital of Wuhan University, Wuhan, China

**Keywords:** Kidney stones, Gut microbiota, Gut microbiota dysbiosis, Diet, Microbiome, Systematic review and meta-analysis

## Abstract

**Background:**

Mounting evidence indicates that the gut microbiome (GMB) plays an essential role in kidney stone (KS) formation. In this study, we conducted a systematic review and meta-analysis to compare the composition of gut microbiota in kidney stone patients and healthy individuals, and further understand the role of gut microbiota in nephrolithiasis.

**Results:**

Six databases were searched to find taxonomy-based comparison studies on the GMB until September 2022. Meta-analyses were performed using RevMan 5.3 to estimate the overall relative abundance of gut microbiota in KS patients and healthy subjects. Eight studies were included with 356 nephrolithiasis patients and 347 healthy subjects. The meta-analysis suggested that KS patients had a higher abundance of *Bacteroides* (35.11% vs 21.25%, Z = 3.56, *P* = 0.0004) and *Escherichia_Shigella* (4.39% vs 1.78%, Z = 3.23, *P* = 0.001), and a lower abundance of *Prevotella_9* (8.41% vs 10.65%, Z = 4.49, *P* < 0.00001). Qualitative analysis revealed that beta-diversity was different between the two groups (*P* < 0.05); Ten taxa (*Bacteroides, Phascolarctobacterium, Faecalibacterium, Flavobacterium, Akkermansia, Lactobacillus, Escherichia coli, Rhodobacter* and *Gordonia*) helped the detection of kidney stones (*P* < 0.05); Genes or protein families of the GMB involved in oxalate degradation, glycan synthesis, and energy metabolism were altered in patients (*P* < 0.05).

**Conclusions:**

There is a characteristic gut microbiota dysbiosis in kidney stone patients. Individualized therapies like microbial supplementation, probiotic or synbiotic preparations and adjusted diet patterns based on individual gut microbial characteristics of patients may be more effective in preventing stone formation and recurrence.

**Supplementary Information:**

The online version contains supplementary material available at 10.1186/s12866-023-02891-0.

## Background

With an estimated 5-year recurrence rate of up to 50% [1], kidney stone (KS) disease is a widespread issue that affects roughly 10–15% of the global population; its prevalence is the highest among urological diseases [2]. The most common kidney stone type is calcium oxalate (76%), followed by hydroxyapatite (18%), uric acid (4.8%), struvite (0.9%), and brushite (0.9%) [3]. Long-term renal stones can cause urinary tract obstruction, infection and irreversible renal function damage, seriously affecting the quality of life and health of patients. Moreover, kidney stone disease increases the risk of chronic kidney disease, end-stage kidney disease, and dialysis therapy [4, 5]. It is believed that environmental, dietary, hormonal, and genetic factors all play a role in the underlying etiology in the majority of patients [6–8]. Symptomatic kidney stone management has evolved from open surgical lithotripsy to minimally invasive endoscopic treatment, which has become the mainstream treatment. However, the demand for more effective therapies to prevent the occurrence and recurrence of stones persists and necessitates a deeper comprehension of the mechanisms underlying stone formation [9].

Recent research has shown a growing connection between human diseases and the microbiota, and this connection is especially strong for the gut microbiome (GMB). GMB is formed by the trillions of commensal bacteria, fungi, archaea and viruses that are densely distributed in the gastrointestinal tract of mammals [10]. Dysbiosis of the GMB can cause metabolic, immune and mental disorders such as inflammatory bowel diseases (IBD), diabetes, chronic kidney disease (CKD), autoimmune arthritis and depression [11–13]. There are several ways to ascertain the composition and diversity of the GMB due to the development of next-generation sequencing technology. For example, DNA microarrays, fluorescence in situ hybridization, and sequencing of 16S rRNA gene or its amplicons are all methods based on sequence divergences in small subunit ribosomal RNA [14, 15]. Metagenomic or shotgun sequencing is another method that randomly sequences all DNA extracted from the sample, which provides more accurate taxonomic resolution, information about the potential function of the microbiome, as well as identification of microbial taxa at the species level [16, 17]. By using shotgun sequencing on patients’ feces, Qian et al. [18] developed the first gut microbial gene catalog as diagnostic biomarkers of Parkinson's disease. The gut microbial dysbiosis and the underlying pathogenesis of diseases such as diabetes, IBD and tuberculosis were further revealed through multi-omics sequencing [19]. Notably, the development of microbial sequencing contributes to the development of prediction, diagnosis and treatment of human diseases.

A close pathogenic association exists between the intestinal microbiota and kidney diseases (gut-kidney axis) like chronic kidney disease, acute kidney injury, glomerulonephritis, nephrotic syndrome, and IgA nephropathy, among others [20]. Patients with CKD, IgA nephropathy or gout have a characteristic GMB dysbiosis [21–23]. Several studies have actively performed gut microbiota sequencing analysis in patients with stones and have established that the gut microbiota is closely associated with stone formation. However, the characteristics of GMB dysbiosis in patients with stones remain unclear due to variant sample sizes, analysis methods and individual differences. For the first time, we analyze the altered gut microbiota composition in patients with kidney stones by conducting a meta-analysis. In addition, we qualitatively summarized the altered diversity, genetic functions and detection capability of GMB in KS patients and discussed the role of diet in the gut-nephrolithiasis axis, as well as promising therapies based on the modulation of gut microbiota to provide potential strategies for the prevention and treatment of kidney stones.

## Methods

This systematic review and meta-analysis were conducted following the Preferred Reporting Items for Systematic Reviews and Meta-Analyses (PRISMA) guidelines and registered at PROSPERO (No. CRD42022367346).

### Search strategy and study selection

Two members of our team independently searched scientific databases namely PubMed, Scopus, Medline, CINHAL, Web of Science, and the Cochrane Library from their inception through September 2022, in consultation with a sciences research librarian. Full search terms and an example of the search strategy can be found in (Supplementary Table 1). Additional articles were searched for in the reference lists of the included studies, systematic reviews, and meta-analyses.

Two members screened the titles and abstracts independently and read the full text of potentially relevant articles carefully. Articles were included if (1) they were original research that examined the gut microbiota from kidney stone patients and compared that to controls; (2) they reported on the microbial community from stool samples. We excluded studies if (1) they were animal or in-vitro studies, commentaries, reviews, meta-analyses or editorials; (2) they detected microbiome from urine or stones samples; (3) they included populations having received chemotherapy within the previous year, or reporting antibiotic use recently.

### Outcome measures

The primary outcome was differences in the abundance of gut microbiota between KS patients and healthy subjects at the genus level. The secondary outcomes were descriptions of the gut microbial diversity, potential detection capability and genetic functions in each study.

### Data extraction and quality assessment

The following information was extracted from the included articles by two independent reviewers: the first author's name, the publication year, the nation, the sample size, microbiota species, statistically significant (*P* < 0.05) relative abundance at the taxonomic level of the gut microbiota between the groups. Disagreements were discussed until a consensus was reached. Alternatively, a third reviewer was able to fix them.

The Newcastle–Ottawa Scale (NOS) was used to assess the quality of the studies [24]. The scale contains eight elements to assess the selection, comparability and identification of exposure of the groups. With a total score of 9, studies were rated as low quality (0–5) or high quality (6–9); low quality studies were excluded.

### Statistical analysis

For continuous outcomes, the relative abundance of gut microbiota, we collected the aggregate mean, standard deviation (SD) and standard error (SE) for quantitative synthesis to obtain the overall percentage of different microbial genera in KS patients and the controls. We also contacted the authors of original studies to obtain the data not available in the article. The relative abundance of gut microbes in kidney stone patients compared to healthy subjects was determined by calculating the difference in percentage of several genera between the KS and the control group. For the diversity, potential detection capability and genetic functions of gut microbiota, we only conducted qualitative synthesis because of the small sample sizes and the insufficient data.

All statistical analyses in this study were performed using the RevMan5.3 statistical software with the inverse-variance method. Forest plots represented the summary metrics. The I^2^ statistic was used to measure statistical heterogeneity between studies [25]. Sensitivity analysis was performed by excluding one study at a time to ensure the stability and accuracy of the study. The funnel plot and begg's test were utilized to evaluate publication bias, even though there might only be a few studies included in subgroup analysis. *P* < 0.05 was considered statistically significant [26].

## Results

### Study selection, characteristics and quality of included studies

**Fig. 1:**
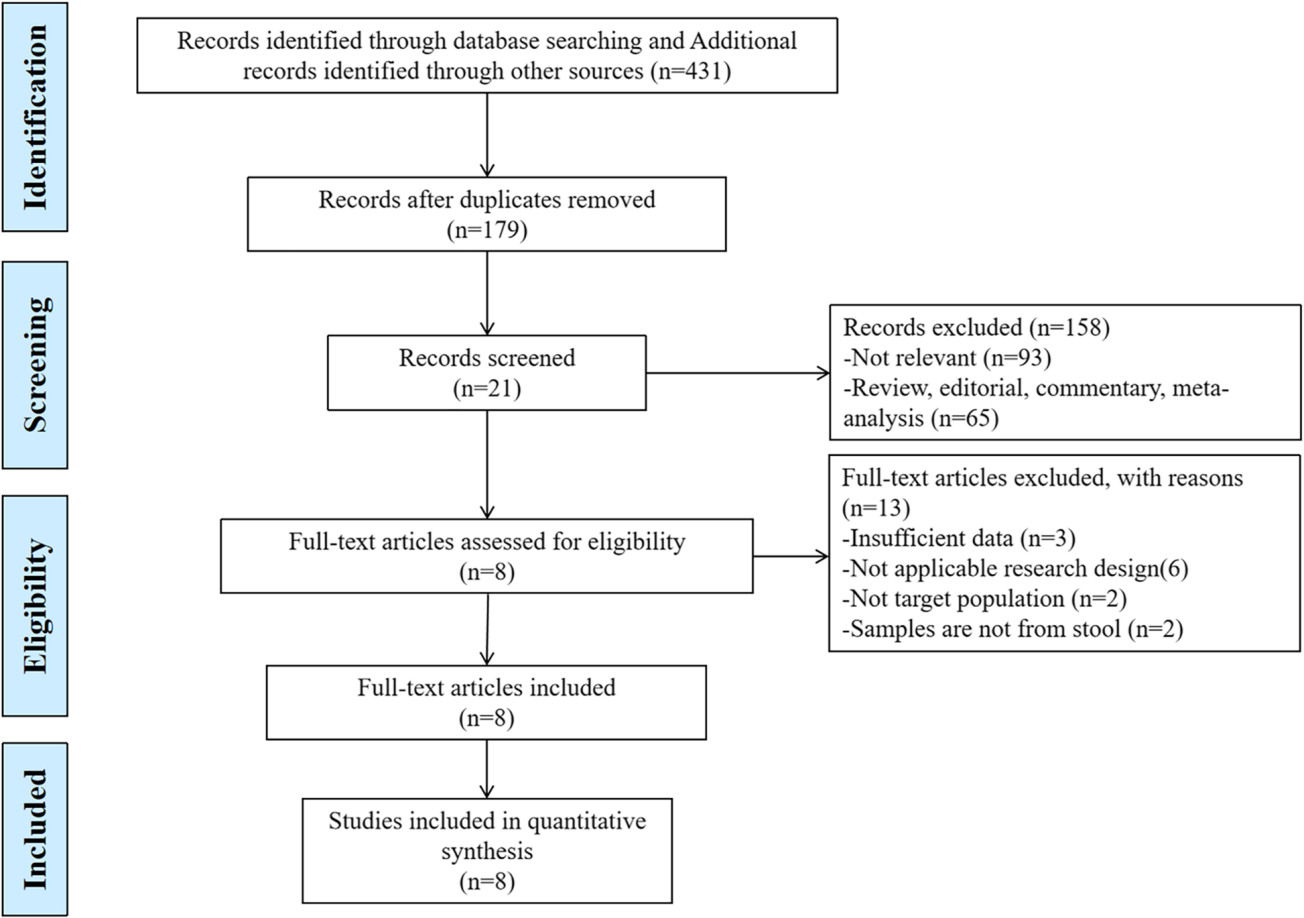
PRISMA flow diagram

A total of eight articles [27–34] were included. The selection process of studies is depicted in Fig. 1. Studies were mainly from China, the USA, Italy and India and account for 703 samples (356 stone patients and 347 controls). All eight studies were observational cohort studies. All eligible studies used stool samples for gut microbiota analysis via high-throughput molecular approaches by Illumina platforms (MiSeq, NextSeq, HiSeq). In addition to 16S rRNA sequencing, two studies combined shotgun sequencing [30], frc-gene amplicon sequencing and denaturing gradient gel electrophoresis (DGGE) fingerprinting [33] to characterize microorganisms. Three studies [28, 29, 34] failed to report α and β diversity. Intestinal flora differed between patients and controls in the included studies, but the specific genera were highly variable and listed in Table 1. All eight of the articles received a NOS score of seven or higher, indicating high quality (Supplementary Table 2). The funnel plot and begg’s test suggested there were no publication bias for included studies (*P* for begg’s test = 0.734) (Supplementary Fig. 4).

**Table 1:** Characteristics of included studies

Reference	Country	KS(n)	HA(n)	Microbiota analysis technique	Diversity metrics		Differentially distributed species	
					α-diversity	β-diversity	Higher abundance in KS group	Higher abundance in control group
Tang et al. 2018 [27]	China	13	13	16S rRNA gene amplicon sequencing (V4 region) Illumina Hiseq 2500 platform	Not significantly different (Chao1, Shannon, Simpson and Good’s coverage indices, *P* > 0.05);	Distinct but not significantly different (NMDS, *p* = 0.096 with ANOSIM and *p* = 0.058 with MPRR based on the Bray–Curtis distance metric)	*Alloprevotella, Erysipelatoclostridium, unidentified_Lachnospiraceae, Phascolarctobacterium, Megamonas, Acinetobacter, Escherichia–Shigella, Sutterella*	*Eubacterium_hallii_group, Dorea, Ruminiclostridium_5, Anaerostipes, Fusicatenibacter, Subdoligranulum, Eubacterium_ruminantium_group, Holdemania, Dialister, Ruminococcus_1, Parasutterella Biliphil*
Stern et al. 2016 [28]	USA	23	6	16S rRNA gene sequencing (V4 region) Illumina MiSeq	Not reported		*Bacteroides, Parabacteroides, Alistipes*	*Varibaculum, Prevotella, Finegoldia, Peptoniphilus*
Zhao et al. 2021 [29]	China	91	75	16S rRNA gene sequencing (V4 region) Illumina MiSeq	Not reported		*Bacteroides, Prevotella*	*Lachnoclostridium, Blautia, Bifidobacterium*
Ticinesi et al. 2018 [30]	Italy	52	48	16S rRNA gene sequencing (V3 region) Illumina MiSeq; Shotgun metagenomics on sub-sample Illumina NextSeq	Significantly reduced (Chao1 index, *P* < 0.05)	Significantly different (PCOA method based on weighted unifrac and Bray–Curtis, *P* < 0.05)	*Bacteroides, Acidaminococcus, Cellulosimicrobium*	*Klebsiella, Enterobacter, Enterorhabdus, Enterococcus, Veillonella, Christensenellaceae R-7 group, Oribacterium, Ruminococcaceae UCG-003, Lachnospiraceae NK4A136 group, Rothia, Family XIII UCG-001, Lachnospiraceae, Ruminiclostridium 5,*
Chen et al. 2021 [31]	China	30	30	16S rDNA gene sequencing (V3 and V4 region) Illumina MiSeq;	Not significantly different (OTUs, Shannon and Simpson index, *P* > 0.05);	Significantly different (PCA, *P* < 0.01)	*Bacteroidetes* and *Phascolarctobacterium*	*Firmicutes, Akkermansia, Faecalibacterium, Lactobacillus* and *Verrucomicrobia*
Yuan et al. 2022 [32]	China	69	84	16S rRNA gene sequencing (V3 and V4 region) Illumina MiSeq PE300 platform	Shannon index was not significantly different (*P* > 0.05), Simpson index was significantly elevated in LNR_KS *vs* HNR_NS group, Ace and Chao index were significantly elevated in the HNR_KS *vs* NS group (*P* < 0.05);	Significantly different between HNR-KS and HNR-NS groups, and between HNR-KS and LNR-NS groups (*P* < 0.05)	Not reported	
Suryavanshi et al. 2016 [33]	India	24	25	16S rRNA gene (V3 region) and frc-gene amplicon sequencing; Quantitative polymerase chain reaction & PCR-DGGE Iron Torrent PGM system	Not significantly different (Chao1, observed species/OTUs, phylogenetic diversity, Shannon and Simpson’s indices, *P* > 0.05);	Significantly different (PCOA plots of weighted and unweighted unifrac results; statistical test and *P*-value not reported)	Phylum level: *Firmicutes, Proteobacteria, TM7;* Class level*: Bacilli, Gammaproteobacteria and TM7-3*	Phylum level: *Bacteroidetes, Cyanobacteria;* Class level: *Bacteroidia, Betaproteobacteria, Chloroplast*, *Coriobacteriia*
Xiang et al. 2022 [34]	China	54	66	16S rRNA gene sequencing	Not reported		*Flavobacterium, Rhodobacter, Gordonia*	

*Abbreviations*: *PCoA* Principal coordinate analysis, *NMDS* Non-metric multi-dimensional scaling, *PCA* Principal component analysis

### Relative abundance of gut microbiota composition

#### Bacteroides

**Fig. 2:**
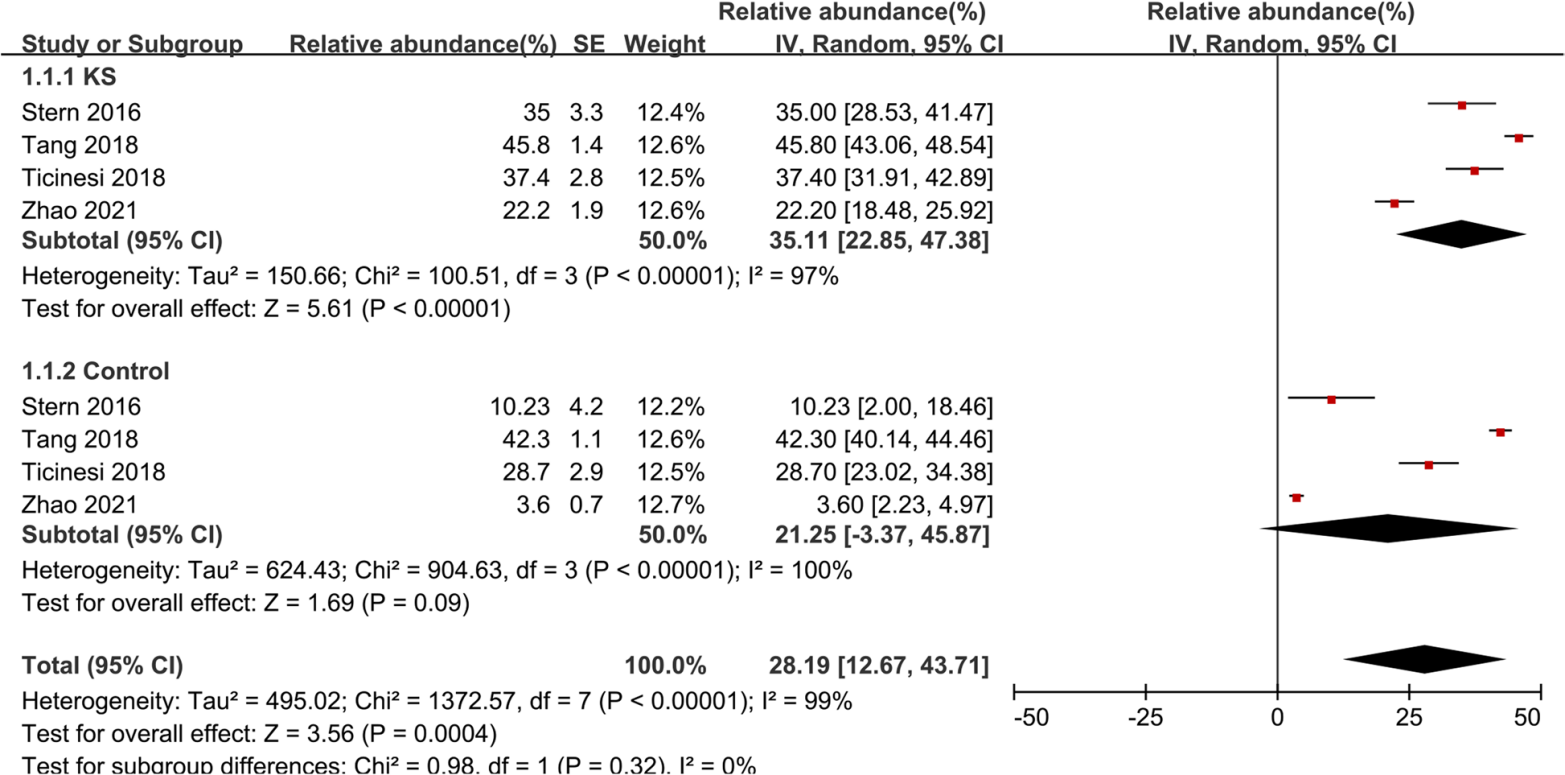
Forest plot of relative abundance of *Bacteroides* in kidney stone patients relative to controls

The random-effects meta-analysis of *Bacteroides* was conducted using data from four studies [27–30] and revealed the following abundance: 35.11% (95% CI, 22.85–47.38, I^2^ = 97%) in KS patients and 21.25% (95% CI, -3.37–45.87, I^2^ = 100%) in the control group (Fig. 2). The overall effect size was moderate and significant (Z = 3.56, *P* = 0.0004), indicating that *Bacteroides* was more abundant in KS patients, the percentage of the KS group was 1. 65-fold higher compared to the control group. Sensitive analysis was done due to obvious heterogeneity (Supplementary Fig. 1).

#### Escherichia_Shigella

**Fig. 3:**
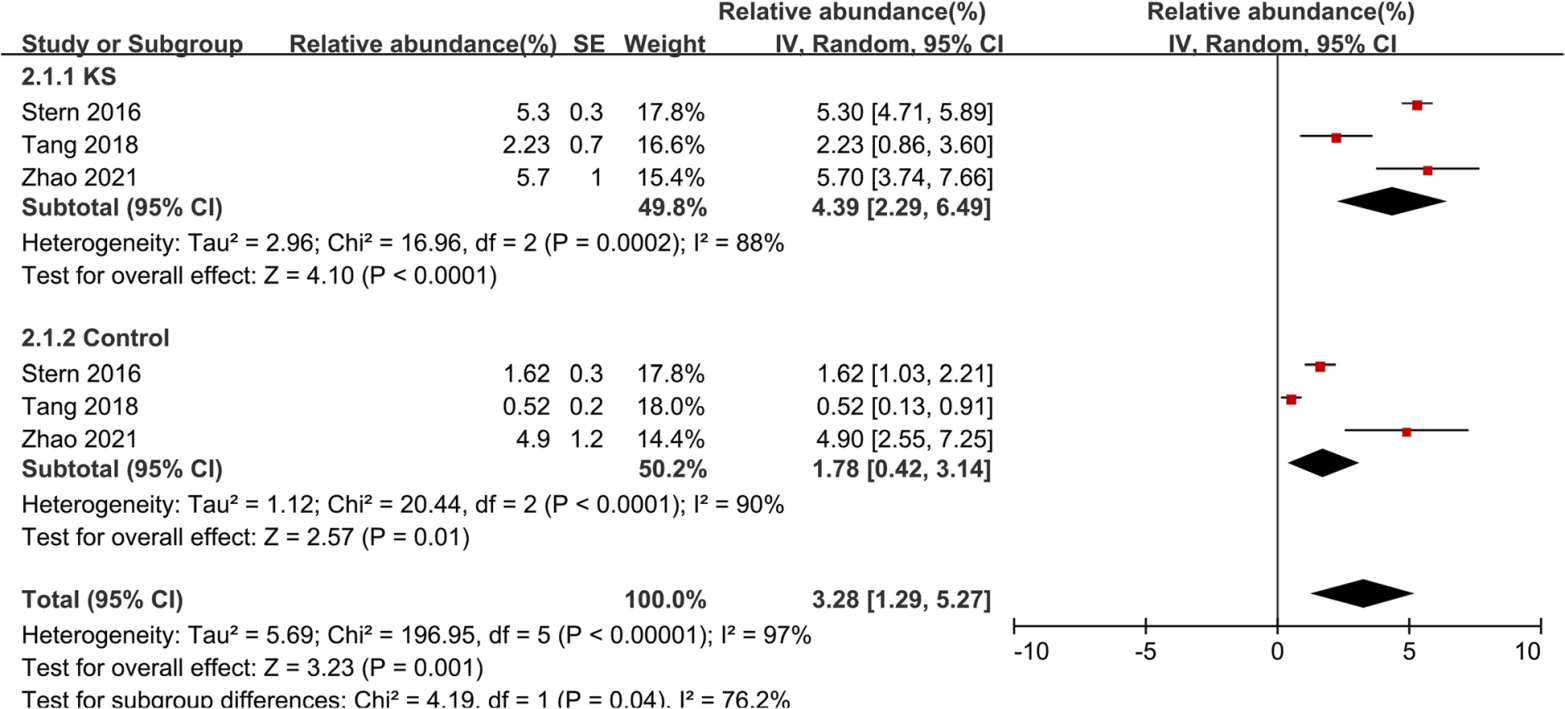
Forest plot of relative abundance of *Escherichia_Shigella* in kidney stone patients relative to controls

Three studies [27–29] were included in the meta-analysis of *Escherichia_Shigella* (Fig. 3). In the KS group, *Escherichia_Shigella* made up 4.39% of the total microbiota detected (95% CI, 2.29–6.49, I^2^ = 88%), while in the control group, it made up 1.78% (95% CI, 0.42–3.14, I^2^ = 90%). The overall effect size was moderate and significant (Z = 3.23, *P* = 0.001), showing that *Escherichia_Shigella* was more abundant in the KS patients, the percentage of the KS group was 2.47-fold higher compared to the control group. Sensitive analysis was done due to obvious heterogeneity (Supplementary Fig. 2).

#### Prevotella_9

**Fig. 4:**
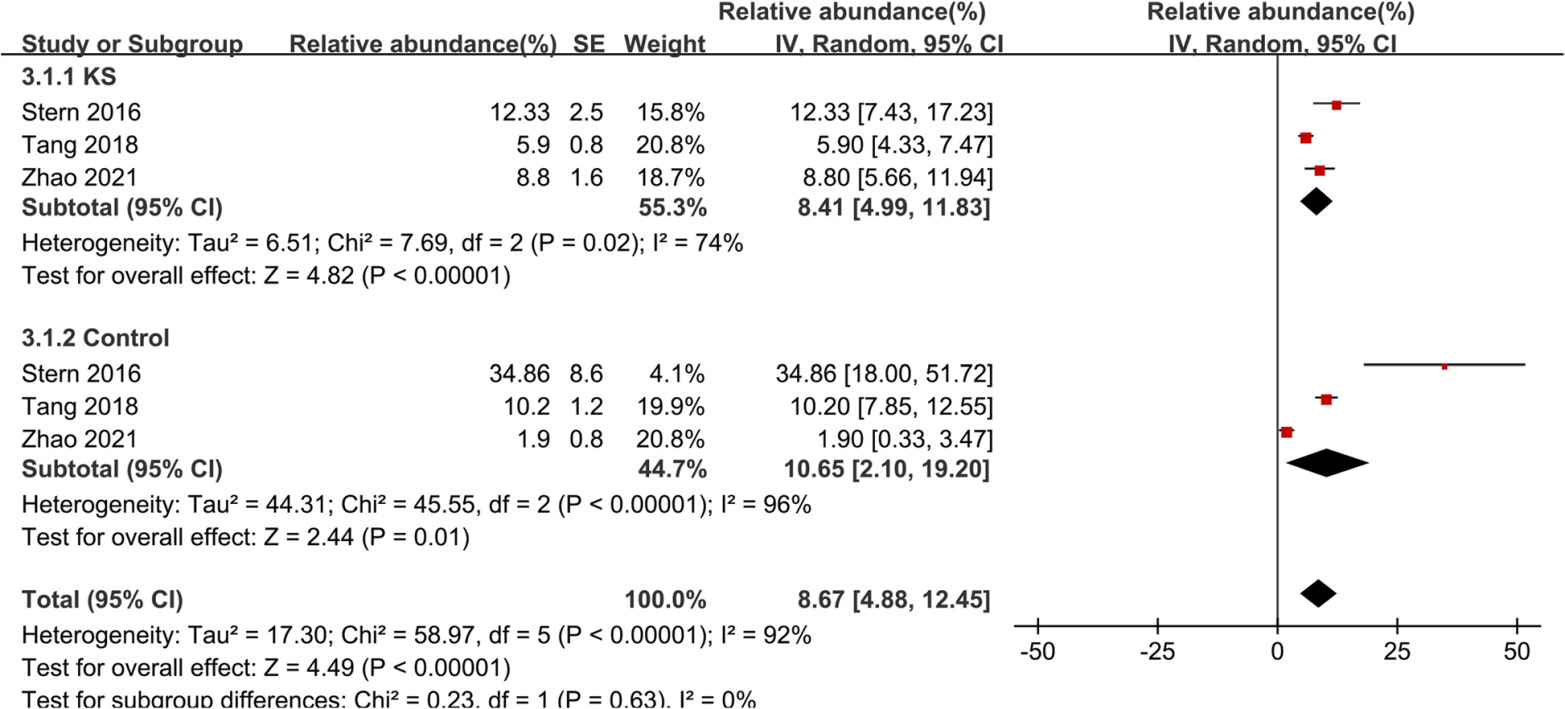
Forest plot of relative abundance of *Prevotella_9* in kidney stone patients relative to controls

The meta-analysis of *Prevotella_9* included three studies [27–29] (Fig. 4). In the KS group, *Prevotella_9* accounted for 8.41% of the total microbiota detected (95% CI, 4.99–11.83, I^2^ = 74%), and in the control groups, it accounted for 10.65% (95% CI, 2.10–19.20, I^2^ = 96%). The overall effect size was moderate and significant (Z = 4.49, *P* < 0.0001), showing that *Prevotella_9* was less prevalent in KS patients than in controls, the percentage of the KS group was 0.77-fold lower compared to the control group. Sensitive analysis was done due to obvious heterogeneity (Supplementary Fig. 3).

### Diversity and richness of gut microbiota in KS patients compared to controls

The number (richness) and distribution (evenness) of taxa within a sample are measured by alpha diversity [35]. Inconsistent results of α-diversity were reported in five articles [27, 30–33], of which three [27, 31, 33] observed no significant difference between the KS and control groups. One study [30] reported that α-diversity (Chao1 index) was significantly reduced in patients with stones (*P* = 0.02). Yuan et al. [32] reported increased α-diversity in stone patients relative to controls (*P* < 0.05), they found that in the KS group, the Simpson (*P* = 0.026), Ace (*P* < 0.01) and Chao (*P* < 0.05) indices were remarkably higher, while Shannon’s index was not significantly different from the control group (*P* > 0.05).

Beta diversity is a diversity indicator that shows how different or similar the microbial compositions of multiple samples are [35]. Five articles [27, 30–33] reported results for β-diversity by PCA, PCoA or NMDS based on Bray–Curtis, weighted and unweighted UniFrac distance metrics. Of the five, four studies [30–33] reported obviously compositional differences of the overall microbial communities between kidney stone patients and controls (*P* < 0.05).

### Potential detection of kidney stones on gut microbiota characteristics

Three studies used receiver-operating characteristic curve (ROC) analysis to identify whether the gut microbiota could be used to distinguish between the two groups. Five genera of the gut microbiota were found to be biomarkers for calcium oxalate stones by Chen et al. [31]: *Bacteroides, Phascolarctobacterium, Faecalibacterium, Akkermansia*, and *Lactobacillus* (AUC = 0.871 CI, 0.785–0.957). According to Tang et al. [27], P. aeruginosa (AUC = 0.947) and *Escherichia coli* (AUC = 0.840) could be used to accurately categorize patients with nephrolithiasis. Xiang et al. [34] reported that the relative abundances of genera *Flavobacterium, Rhodobacter, Gordonia* were found useful in predicting kidney stones (AUCs ranging from 0.682 to 0.763). They found that using data from the three genera and four clinical indicators (oxalate concentration, acetic acid concentration, citrate concentration, phosphorus concentration) together produced predictions that were more accurate than those made using just genus or clinical data, and random forest developed the most accurate model (AUC = 0.936).

### Altered genetic functions of the gut microbiota in KS patients

Four studies reported using shotgun sequencing, functional gene-targeted amplicon sequencing, or the bioinformatics software platform PICRUSt that the predicted genetic functions of KS patients were different from the controls. Through shotgun metagenomics, Ticinesi et al. [30] performed a functional analysis of the fecal microbiota, revealing a decreased representation of genes involved in oxalate degradation in patients compared to controls (0.0021% vs 0.0041% of the total genome, *P* = 0.007), such as formyl-CoA transferase and oxalyl-CoA decarboxylase. Interestingly, these results were challenged by Suryavanshi et al. [33] who observed that the enzymes involved in oxalate degradation were enriched in KS patients. Tang et al. [27], reported observing that the oxalate degradation enzymes were not statistically different from healthy controls to nephrolithiasis subjects (*P* > 0.05).

Other different functions including glycan synthesis, energy metabolism, co-factors metabolism, and vitamins metabolism, of which protein families were downregulated in KS patients according to Suryavanshi et al. [33], whereas protein families of lipid metabolism, carbohydrate metabolism and xenobiotic degradation and metabolism were up-regulated in KS subjects (*P* < 0.05). One study indicated that, compared to healthy subjects, a higher abundance of metabolic pathways related to inflammation, lipid metabolism, xenobiotics biodegradation and mineral metabolism were found in KS patients (*P* < 0.05) [32].

## Discussion

To our knowledge, this is the first meta-analysis of the gut microbiota composition of patients with kidney stones. There are consistent results indicating a characteristic gut microbiota dysbiosis in kidney stone patients. Identifying these dysbiosis characteristics may help in personalized clinical intervention. The results showed that, compared to healthy subjects, those with nephrolithiasis showed a higher relative abundance of genera *Bacteroides* and *Escherichia_Shigella*, and a lower relative abundance of genera *Prevotella_9*. We could not conduct meta-analysis of the altered distribution of other gut microbial genera due to insufficient data and limited overlap of findings. A clinical study reported that the expansion of *Escherichia_Shigella* in gut was related with the onset and response to immunosuppressive therapy of IgA nephropathy, and may serve as a promising diagnostic biomarker and therapeutic target [23]. *Escherichia* trended to an inverse correlation with urinary citrate (*r* =  − 0.56, *P* < 0.08) [28], which can bind free calcium and create soluble calcium citrate, lowering the supersaturation of calcium-containing salts, making it a urinary stone inhibitor [36]. With the decline of citrate concentration, the risk of nephrolithiasis increased. *Bacteroides* were found useful in detecting kidney stone disease [31]. Some *Bacteroides* can produce urease [37], which degrades urea and promotes the formation of ammonia and carbon dioxide, leading to urine alkalinization and phosphate salt formation. It has been reported that the altered gut microbial species have been associated with various clinical parameters: the abundance of *Bacteroides* has a negative relationship with all short-chain fatty acids (SCFAs) [31]; the abundance of *Prevotella* negatively correlated with urine oxalate content [33] and is positively correlated with serum uric acid levels [29]. Modulating the relative abundance of gut microbiota based on the dysbiosis characteristics may be an effective therapeutic strategy for nephrolithiasis prevention and treatment.

Numerous other clinical disorders, such as inflammatory bowel disease (IBD), obesity, type 1 and type 2 diabetes, have been linked to lower gut microbial diversity [38, 39]. However, differences in gut microbial diversity between kidney stone groups and controls remain inconclusive in this review. The β-diversity analysis of the four studies showed statistically significant difference between kidney stones and the control group, which indicated that the microbial community structure was significantly different. However, the results of α-diversity measurements were inconsistent among the eight studies. One study reported a significant reduction in α-diversity in patients with kidney stones compared to controls, while another one reported increased gut microbial α-diversity in stone patients relative to controls. Studies have shown that decreased α-diversity of gut microbiota may increase the risk of stone formation by reducing immunity and the ability to metabolize oxalate, phosphate, and citrate; an increase in microbial abundance in patients may reflect the proliferation of some potential pathogenic bacteria species, through the relevant intestinal uric poisonous toxin concentration increase, leading to the loss and destruction of intestinal epithelial barrier [40], so as to promote the occurrence and progress of kidney disease. It may be due to the individual differences, insufficient sample size and different control of the confounding factors of the original study that lead to the inconsistent results of diversity. More studies with larger sample sizes and comparable and reproducible methods are required to determine the changes in intestinal microbial diversity in stone patients.

Advanced omics methods have enabled unprecedented levels of functional characterization and deep sequencing of gut microbial communities over the past ten years [41]. The systematic reviews suggested that functional activities of the gut microbiota involved in oxalate degradation, lipid, carbohydrate and energy metabolism, glycan synthesis and amino acid biosynthesis were altered in KS patients compared to controls. The studies established that the gut-nephrolithiasis axis is not limited to *Oxalobacter formigenes* [30, 33]. It has been proved that urinary oxalate is a crucial risk factor for kidney stones [42, 43]. The intestinal tract plays a significant role in oxalate balance and subsequent oxalate homeostasis [44, 45]. Gut microbiota with oxalate degradation properties (*Oxalobacter, Bifidobacterium, and Lactobacillus*) may inhibit stone formation through extra-renal elimination of oxalate in the intestines and decreasing the oxalate concentration in plasma and urine [46]. Genes involved in lipid metabolism were enriched in stone patients, which indicated that dysbiosis of gut microbiota may contribute to stone formation by promoting lipid metabolism. Several studies have suggested that some fatty acid supplements improve gut microbial diversity and thus improve diseases including nervous system disorders and alcoholic liver disease [47, 48], and N-3 fatty acid supplementation has been shown to reduce the levels of important stone risk factors, namely, hypercalciuria [49–51], hyperoxaluria [52]. Glycan synthesis functions of altered gut microbiota were observed to be downregulated. Hyperglycemia may contribute to the development of nephrolithiasis [53] by changing hydro-electrolytes in 24-h urine excretions, which leads to more acidic urine, a problem with renal acid excretion, and hypo-citraturia [54], all of which are significant risk factors for stone formation. Carbohydrate metabolism disorder caused by gut microbiota dysbiosis delivers a marked acid load to the kidney, might decrease calcium balance and increase the risk for stone formation [55]. Overall, the predicted gene functional profile suggests that dysbiosis of gut microbiota may contribute to stone formation by breaking the metabolic homeostasis, and may be considered for developing new therapeutic strategies. At the meantime, to further clarify the relationship of the gut microbiota, host metabolism and stone formation, more thorough investigations using shotgun metagenomics, metatranscriptomics, and metaproteomics are required.

The metagenome-wide association study conducted by Zhernakova et al. has shown that inter-individual variations in gut microbiota composition are linked to a number of factors, including lifestyle, diet, diseases, and medications, of which dietary factors are the most crucial [56]. Dietary patterns like high tea consumption reduce the abundance of *Akkermansia spp.* and *Lactobacillus spp.* to promote the formation of calcium oxalate renal calculi [31]. Shikany’s team [57] reported the *Prevotella* enterotype to be associated with low nephrolithiasis risk dietary patterns characterized by a high-carbohydrate diet, while the *Bacteroides* enterotype was associated with high nephrolithiasis risk dietary patterns characterized by a high intake of animal protein [58]. Several studies have shown that high fiber intake also modulates the composition of the gut microbiota, increasing the number of the SCFA-producing species *Lactobacillus* and other health-promoting species [59–61], some of which possess characteristics of oxalate degradation. Although few studies have specifically focused on the role of dietary patterns in the gut-nephrolithiasis axis, the stone-promoting effects of high salt, high oxalate intake, and the stone-preventing effects of increasing fruit, vegetable, juice and water intake, are at least in part mediated by altered gut microbiota composition and metabolic function [62]. Therefore, we believe that for high-risk groups, increasing the intake of fruits, vegetables, fiber and carbohydrates, and reducing that of tea and animal protein can prevent the formation and recurrence of stones. However, as nutrients are not consumed in isolation, the complex interplay between dietary factors of overall dietary patterns is far from being fully understood, and further research is needed to address these issues.

**Fig. 5:**
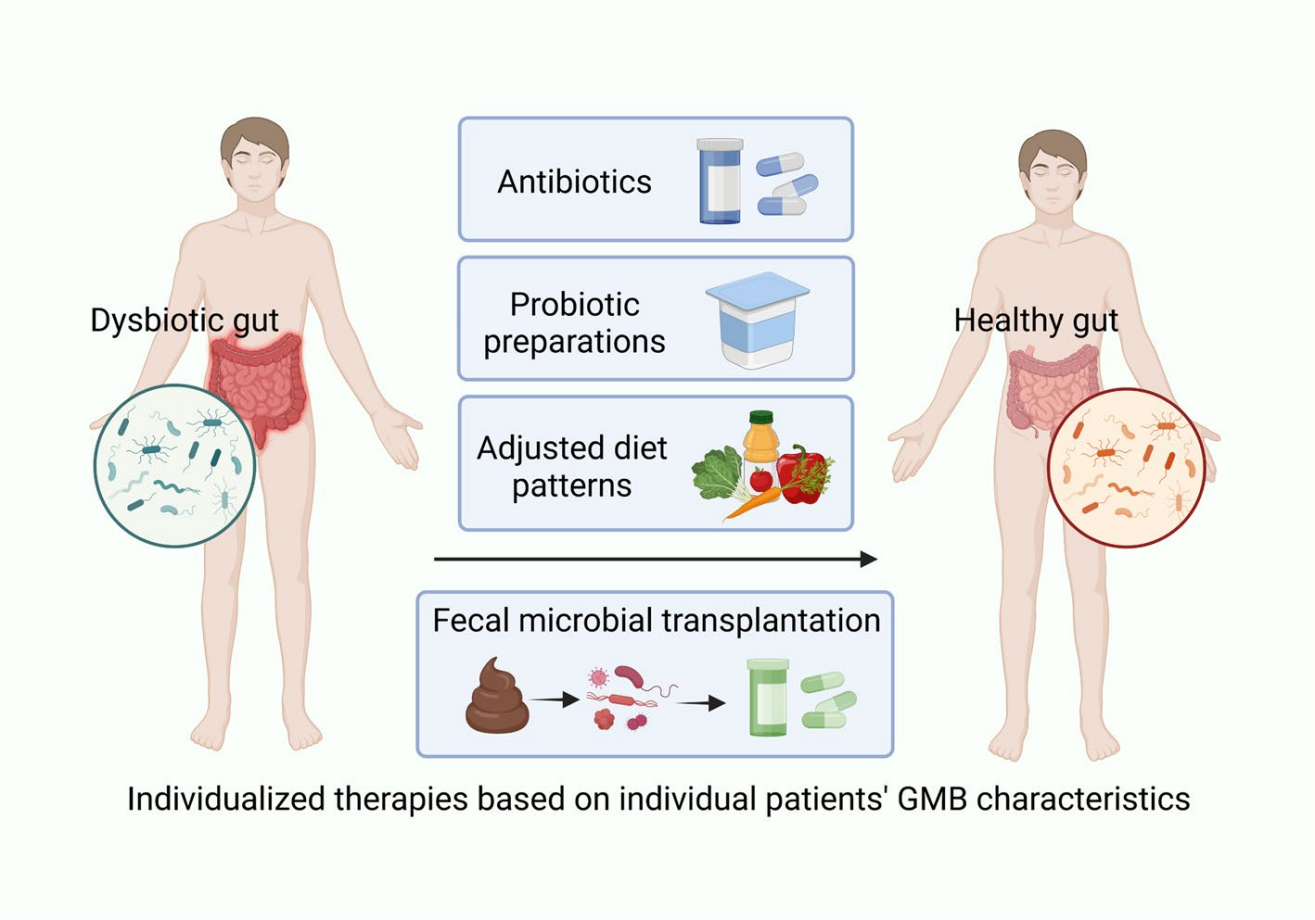
Various methods for restoration of gut microbial dysbiosis to prevent occurrence and recurrence of kidney stones

The above analysis provides us with further insights into the prevention and treatment of kidney stones. Restoring gut dysbiosis to maintain the balance of oxalate, citrate, and lipid metabolism may become a promising treatment for kidney stones. Antibiotics are proven to be effective in gut microbiota modulation by preventing pathogenic bacteria, but they also kill beneficial microbial communities and lead to antibiotic resistance in harmful bacteria [63]. The use of oral probiotic preparations, including *Oxalobacter formigenes, Lactobacillus, Bifidobacterium, Enterococcus*, or other oxalate-lowering bacteria, alone or in combination, resulted in a reduction in urinary oxalate excretion in humans and rodents [64]. Miller et al. [65] recently transplanted oxalate-degrading bacteria into a laboratory rat, showing that whole community microbial transplantation is an effective method to colonize oxalate-degrading bacteria continuously in mammalian intestinal tract. Growing evidence supports *Lactiplantibacillus Plantarum*'s ability to prevent kidney stone formation. Probiotic *Lactiplantibacillus Plantarum* N-1 prevented kidney stones caused by ethylene glycol by regulating the gut microbiota and improving intestinal barrier function [66], *Lactiplantibacillus Plantarum* J-15 corrected metabolic disorder, protected intestinal barrier function, and reduced inflammation to prevent stone formation [67]. Synbiotic supplementation improved the total oxalate-degrading activity of gut microbiota, resulting in decreased oxalate excretion in rats [68]. The safety and efficacy of fecal microbiota transplantation have been demonstrated in the treatment of diseases such as metabolic syndrome, diabetes, cancer and Parkinson's disease [69]. In the future, we will also look forward to personalized fecal microbiota transplantation strategies according to the condition of stone patients. Although most of the current studies are based on animal experiments, the appropriate dietary intervention has been proven a contribution in the prevention of recurrent stones in humans [62]. It may be more effective to take into account the characteristics of GMB of individual patients when formulating individualized therapeutic strategies in future clinical interventions (Fig. 5).

This study is limited in that the number of studies and samples restricts the meta-analysis of more microbial species, and thus, more studies with larger sample sizes in this field are required to obtain more specific data on characteristic dysbiosis. In the included studies, only the effects of chemotherapy and antibiotics on intestinal flora were excluded; confounding variables like food and geography were not taken into account. Some of the included studies did not distinguish between primary and recurrent kidney stone formation and did not distinguish between the types of stones.

## Conclusions

Kidney stone patients have a characteristic gut microbiota dysbiosis with higher relative abundance of *Bacteroides* and *Escherichia_Shigella*, lower relative abundance of *Prevotella_9*. The dysbiotic gut affects the formation of stone by regulating the host metabolism, particularly oxalate metabolism. Gut microbiota characteristics combined with clinical indicators help predict the occurrence and recurrence of kidney stones. High protein, tea, low carbohydrate, juice or fiber intake are risk factors for stone formation. Individualized therapies like fecal microbial transplantation, probiotic or synbiotic preparations and adjusted diet patterns based on the individual gut microbiota characteristics are promising therapeutic options.

## Supplementary Information

**Additional file 1:** **Supplementary Table 1.** An example of the search strategy. **Supplementary Table 2.** Quality assessment of studies included for the meta-analysis. **Supplementary** **Figure 1.** Sensitivity analysis of relative abundance of *Bacteroides* in subgroup of kidney stone patient vs control. **Supplementary** **Figure 2.** Sensitivity analysis of relative abundance of *Escherichia_Shigella* in subgroup of kidney stone patient vs control. **Supplementary Figure 3.** Sensitivity analysis of relative abundance of *Prevotella_9* in subgroup of kidney stone patient vs control. **Supplementary** **Figure 4.** Funnel plot of studies included for meta-analysis.

## Data Availability

The datasets used and/or analyzed during the current study available from the corresponding author on reasonable request.
