# Supplementary material for: Gut microbiota in patients with kidney stones: a systematic review and meta-analysis
Source: BMC Microbiol. 2023 May 19;23:143. doi: 10.1186/s12866-023-02891-0 (PMC10197343; doi:10.1186/s12866-023-02891-0)

Supplementary Material

# Supplementary Table 1. An example of the search strategy.

| **Database/platform:** | **PubMed** |
| --- | --- |
| **Library:** | Free access |
| **Date of search** | 10/2022 |
| **Limits:** | None applied |
| **Search query:** | (("Kidney Calculi"[MeSH Terms] OR ("Kidney Calculi"[MeSH Terms] OR ("kidney"[All Fields] AND "calculi"[All Fields]) OR "Kidney Calculi"[All Fields] OR ("calculi"[All Fields] AND "kidney"[All Fields]) OR "calculi kidney"[All Fields]) OR ("Kidney Calculi"[MeSH Terms] OR ("kidney"[All Fields] AND "calculi"[All Fields]) OR "Kidney Calculi"[All Fields] OR ("calculus"[All Fields] AND "kidney"[All Fields]) OR "calculus kidney"[All Fields]) OR ("Kidney Calculi"[MeSH Terms] OR ("kidney"[All Fields] AND "calculi"[All Fields]) OR "Kidney Calculi"[All Fields] OR ("kidney"[All Fields] AND "calculus"[All Fields]) OR "kidney calculus"[All Fields]) OR ("Kidney Calculi"[MeSH Terms] OR ("kidney"[All Fields] AND "calculi"[All Fields]) OR "Kidney Calculi"[All Fields] OR ("renal"[All Fields] AND "calculus"[All Fields]) OR "renal calculus"[All Fields]) OR ("Kidney Calculi"[MeSH Terms] OR ("kidney"[All Fields] AND "calculi"[All Fields]) OR "Kidney Calculi"[All Fields] OR ("kidney"[All Fields] AND "stones"[All Fields]) OR "kidney stones"[All Fields]) OR ("Kidney Calculi"[MeSH Terms] OR ("kidney"[All Fields] AND "calculi"[All Fields]) OR "Kidney Calculi"[All Fields] OR ("kidney"[All Fields] AND "stone"[All Fields]) OR "kidney stone"[All Fields]) OR ("Kidney Calculi"[MeSH Terms] OR ("kidney"[All Fields] AND "calculi"[All Fields]) OR "Kidney Calculi"[All Fields] OR ("stone"[All Fields] AND "kidney"[All Fields]) OR "stone kidney"[All Fields]) OR ("Kidney Calculi"[MeSH Terms] OR ("kidney"[All Fields] AND "calculi"[All Fields]) OR "Kidney Calculi"[All Fields] OR ("stones"[All Fields] AND "kidney"[All Fields]) OR "stones kidney"[All Fields]) OR ("Kidney Calculi"[MeSH Terms] OR ("kidney"[All Fields] AND "calculi"[All Fields]) OR "Kidney Calculi"[All Fields] OR ("renal"[All Fields] AND "calculi"[All Fields]) OR "renal calculi"[All Fields]) OR ("Kidney Calculi"[MeSH Terms] OR ("kidney"[All Fields] AND "calculi"[All Fields]) OR "Kidney Calculi"[All Fields] OR ("calculi"[All Fields] AND "renal"[All Fields]) OR "calculi renal"[All Fields]) OR ("Kidney Calculi"[MeSH Terms] OR ("kidney"[All Fields] AND "calculi"[All Fields]) OR "Kidney Calculi"[All Fields] OR ("calculus"[All Fields] AND "renal"[All Fields]) OR "calculus renal"[All Fields]))) AND ((((((((((((((Gastrointestinal Microbiomes[MeSH Terms]) OR (Gut Microbiome)) OR (Gut Microbiomes)) OR (Gut Microflora)) OR (Gut Microbiota)) OR (Gastrointestinal Flora)) OR (Gut Flora)) OR (Gastrointestinal Microbiota)) OR (Gastrointestinal Microbial Community)) OR (Gastrointestinal Microbial Communities)) OR (Intestinal Microbiome)) OR (Intestinal Microflora)) OR (Intestinal Flora)) OR (Gastrointestinal Microflora)) |
| **Number of hits** | 111 hits |
| **Notes** |  |

**Supplementary Table 2.** Quality assessment of studies included for the meta-analysis.

| **Studies** | **Quality assessment criteria** | | | |
| --- | --- | --- | --- | --- |
|  | **Selection** | **Comparability** | **Exposure/Outcome** | **Overall quality** |
| Tang et al. 2018 | ** | ** | *** | 7 |
| Stern et al. 2016 | ** | ** | *** | 7 |
| Zhao et al. 2021 | *** | ** | *** | 8 |
| Ticinesi et al. 2018 | *** | ** | *** | 8 |
| Chen et al. 2021 | *** | ** | ** | 7 |
| Yuan et al. 2022 | *** | ** | ** | 7 |
| Suryavanshi et al. 2016 | ** | ** | *** | 7 |
| Xiang et al. 2022 | *** | ** | ** | 7 |

The study quality was assessed according to the Newcastle Ottawa Quality assessment scale for observational studies. ** 2 points. *** 3 points. **** 4 points.

**Supplementary** **Figure 1**. Sensitivity analysis of relative abundance of *Bacteroides* in subgroup of kidney stone patient vs control.


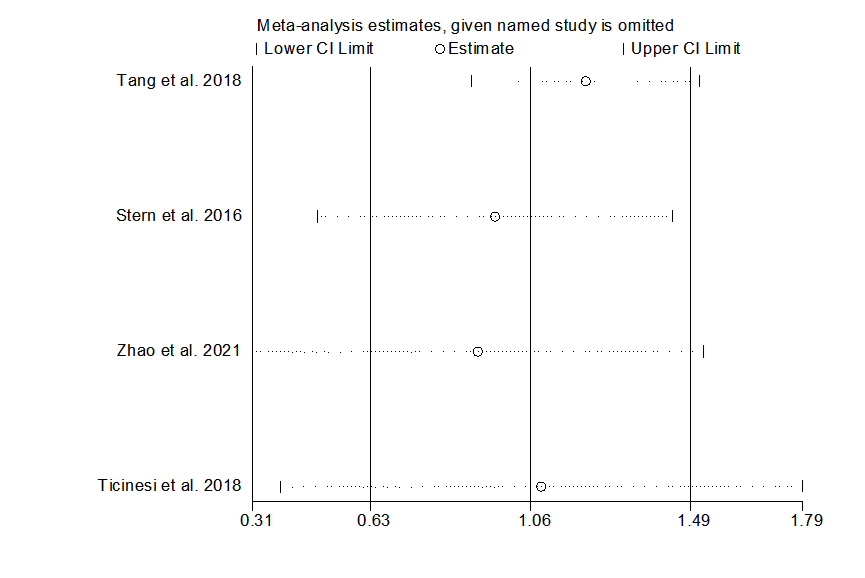


**Supplementary** **Figure 2**. Sensitivity analysis of relative abundance of *Escherichia_Shigella* in subgroup of kidney stone patient vs control.


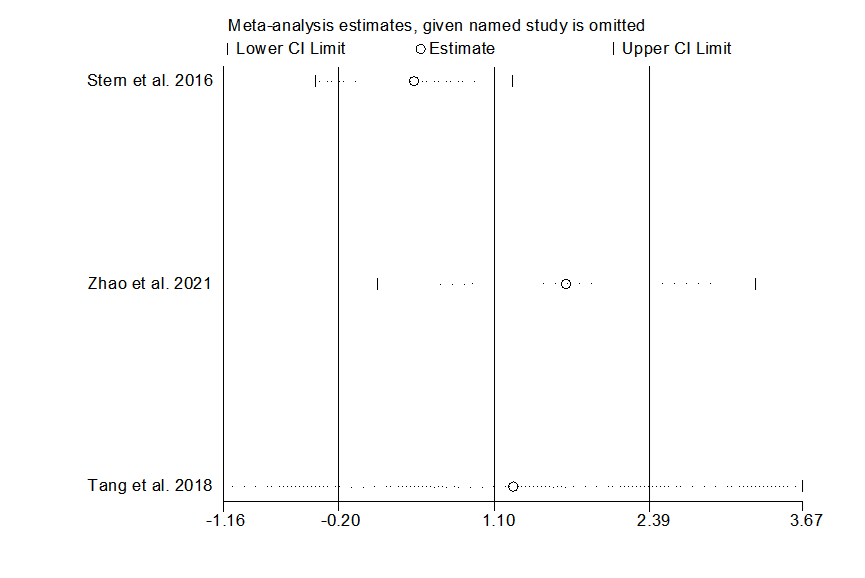


**Supplementary Figure 3**. Sensitivity analysis of relative abundance of *Prevotella_9* in subgroup of kidney stone patient vs control.

**Supplementary** **Figure 4**. Funnel plot of studies included for meta-analysis.


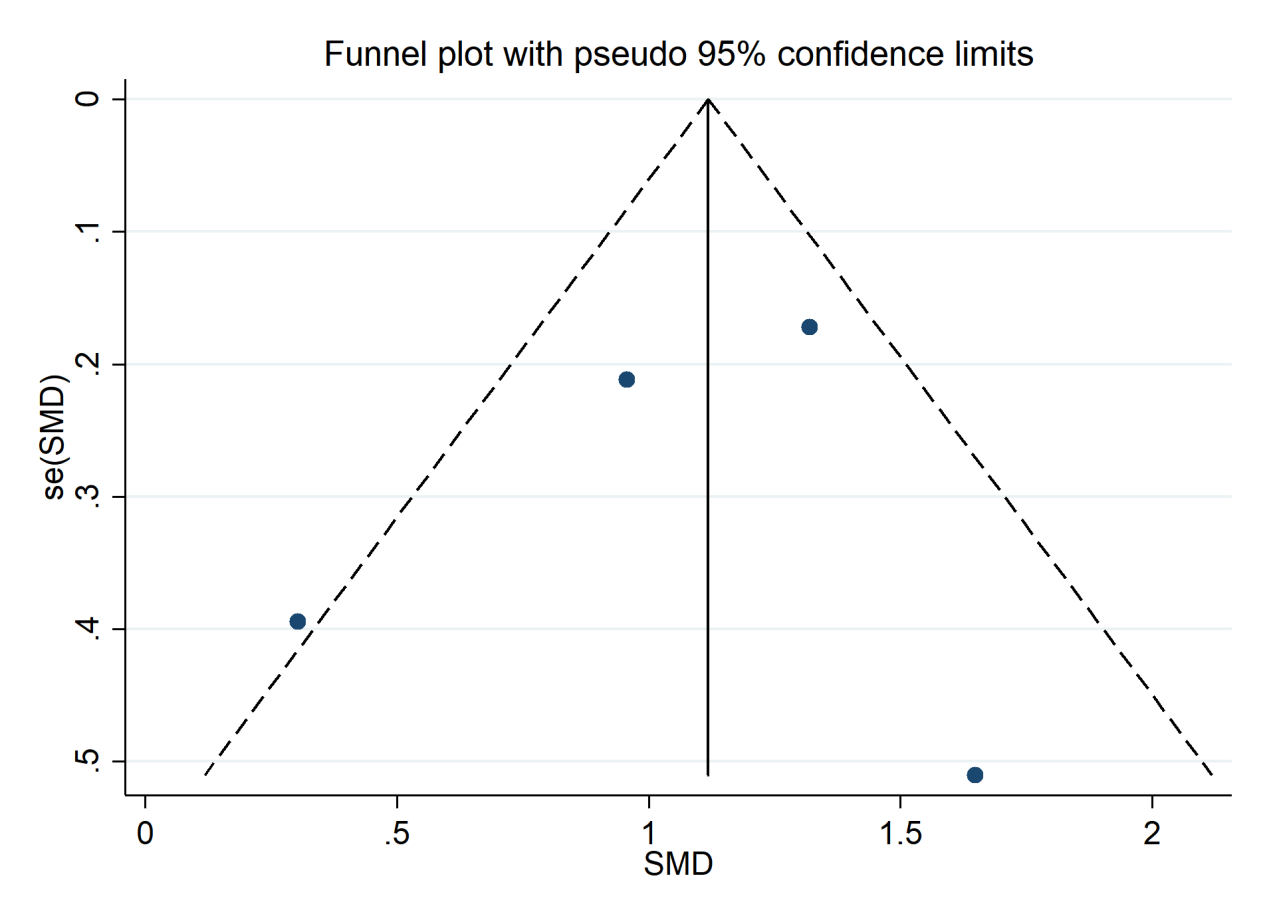

Supplement: Supplementary file 1 — Additional file 1: Supplementary Table 1. An example of the search strategy. Supplementary Table 2. Quality assessment of studies included for the meta-analysis. Supplementary Figure 1. Sensitivity analysis of relative abundance of Bacteroides in subgroup of kidney stone patient vs control. Supplementary Figure 2. Sensitivity analysis of relative abundance of Escherichia_Shigella in subgroup of kidney stone patient vs control. Supplementary Figure 3. Sensitivity analysis of relative abundance of Prevotella_9 in subgroup of kidney stone patient vs control. Supplementary Figure 4. Funnel plot of studies included for meta-analysis. [file 12866_2023_2891_MOESM1_ESM.docx]
